# Is tomosynthesis an ingenious scheme for bronchoscopic diagnosis of lung nodules?

**DOI:** 10.1111/resp.13975

**Published:** 2020-11-17

**Authors:** Taichiro Goto

**Affiliations:** ^1^ Lung Cancer and Respiratory Disease Center Yamanashi Central Hospital Yamanashi Japan

## Abstract

See related Reply

*To the Editors*:

Bronchoscopic diagnosis of small peripheral nodules is still unsatisfactory, requiring improvements in technology and systems.^1^ In a recent publication in *Respirology*, Aboudara *et al*. have reported a new method of fluoroscopic electromagnetic navigation bronchoscopy (F‐ENB) and compared its efficacy and safety with standard ENB (s‐ENB).^2^ They have shown that F‐ENB may increase the diagnostic yield of ENB for small peripheral nodules (s‐ENB: 54.4%, F‐ENB: 79.1%, *P* = 0.0019) with a low complication rate (s‐ENB: 5.9%, F‐ENB: 3.0%).^2^

Tomosynthesis‐assisted navigational bronchoscopy using conventional fluoroscopic C‐arm is a novel scheme that can correct subtle divergence between the catheter tip and the nodule in a two‐dimensional image and achieve correct alignment. The authors obtained an additional oblique image by an easier method and verified the efficacy (diagnostic yield) and safety (low complication rate) of F‐ENB by comparing it to s‐ENB.^2^ F‐ENB, which employs the fluoroscopic C‐arm frequently used in the clinic, appears to be a simple and promising method directly applicable to clinical practice. However, verification of its invasiveness and other improvements is necessary to establish it as a standard diagnostic method.

Because a breath‐hold manoeuvre is required to capture the tomosynthesis image, the F‐ENB uses a neuromuscular blockade, which is associated with the risk of asphyxia. Alternative methods requiring voluntary breath hold by patients should, therefore, be explored in the future. For example, can tomosynthesis images be obtained with patients breathing spontaneously under local anaesthesia and oxygen inhalation, holding their breath for a maximum of 30 s? As for physical invasiveness, the total procedural duration and radiation exposure dose for F‐ENB need to be examined further. In addition, I would recommend that the authors perform a questionnaire‐based survey of the patients to assess the invasiveness of F‐ENB directly.

Recently, computed tomography (CT)‐guided biopsies have often been used instead of bronchoscopy for histological diagnosis of lung tumours. However, it is associated with severe complications, such as air embolism and cancer dissemination.^3^ Thus, improvement in bronchoscopic techniques is essential for a better diagnosis of lung tumours. New technologies including ultrathin fibrescope, navigation system and endobronchial ultrasound; analytical techniques such as DNA analysis of bronchial wash fluid; and new testing systems (ROSE: rapid onsite cytology) have all been developed and improved in the last 20 years.^4^ Besides, many minor innovative modifications of tests used in clinical practice have contributed to better diagnostic yield.^1^ By improving the diagnostic yield by about 25%, the F‐ENB represents a significant evolution in the innovative modifications associated with bronchoscopy. I expect bronchoscopy to achieve a diagnostic yield comparable to that of CT‐guided biopsy. It may be a game‐changer in lung cancer diagnosis, allowing a diagnostic yield comparable to that seen in other cancers (gastric and colorectal).

This study is a retrospective comparative study with historical control. To reduce biases, a multi‐institutional prospective comparative study and randomized controlled trials based on the data from this study should be carried out. Large‐scale trials should be conducted soon to validate the clinical utility of F‐ENB and establish it as a new diagnostic standard.
